# Esophageal Involvement of Immunoglobulin G4-Related Disease: A Case Report and Literature Review

**DOI:** 10.1097/MD.0000000000002122

**Published:** 2015-12-18

**Authors:** Ji Hyun Oh, Tae Hee Lee, Hyo Shik Kim, Chan Sung Jung, Joon Seong Lee, Su Jin Hong, So-Young Jin

**Affiliations:** From the Institute for Digestive Research, Digestive Disease Center, Department of Internal Medicine, College of Medicine, Soonchunhyang University, Seoul (JHO, THL, HSK, CSJ, CSJ, JSL), Institute for Digestive Research, Digestive Disease Center, Department of Internal Medicine, College of Medicine, Soonchunhyang University, Bucheon (SJH), and Department of Pathology, College of Medicine, Soonchunhyang University, Seoul, Republic of Korea (SYJ).

## Abstract

Immunoglobulin G4 (IgG4)-related disease is characterized by the typical histopathological features of a dense lymphoplasmacytic infiltrate rich in IgG4-positive plasma cells, a high ratio of IgG4- to IgG-positive cells, storiform fibrosis (cellular fibrosis organized in an irregular whorled pattern), obliterative phlebitis, and variable presence of eosinophils. The disease exhibits systemic involvement but very rarely involves the esophagus.

A 33-year-old man was admitted to our hospital for evaluation of a 1-year history of progressive dysphagia. Neck imaging revealed a 3.9-cm mass in the cervical esophagus and multifocal calcified lymph nodes in the lower neck and mediastinum. Two previous tertiary hospitals failed to diagnose the patient's condition despite the use of ultrasound-guided needle biopsy of the neck tumor. We performed neck imaging studies, a flexible endoscopic swallowing study, high-resolution manometry, upper endoscopy, and a review of the previous pathologic slides. The patient was finally diagnosed with IgG4-related esophagitis and showed a good response to corticosteroid therapy.

We herein report a rare case of dysphagia associated with IgG4-related disease and present a review of the literature.

## INTRODUCTION

Immunoglobulin G4-related disease (IgG4-RD) is a recently recognized systemic disease of unknown etiology and represents a syndrome characterized by specific pathologic, serologic, and clinical features. It was first described in the context of type 1 autoimmune pancreatitis.^1–3^ The disease is an inflammatory and fibrosing condition that causes tumor-like swelling of involved organs, sometimes mimicking malignancy, and other inflammatory or immune-mediated disorders.^4^ It is often combined with elevated serum IgG4 concentrations.

IgG4-RD has been described in a variety of organs and often involves the pancreas. Extrapancreatic organs are also frequently affected, including the biliary tree, salivary glands, periorbital tissues, kidneys, lungs, lymph nodes, meninges, aorta, breast, prostate, thyroid, pericardium, and skin. However, IgG4-RD is extremely rare in the esophagus.^4,5^

The clinical presentation depends on the involved tissues. However, the histopathologic findings seem to be similar regardless of location, and diagnosis is therefore based on histopathology and immunohistochemistry. Additionally, patients often respond well to corticosteroid and immunosuppressive therapy.^3^ We herein report a rare case of IgG4-RD presenting as a tumor-like esophageal mass that showed a good response to corticosteroid therapy.

## CASE REPORT

### Patient Information, Clinical Findings

A previously healthy 33-year-old man presented with a 1-year history of progressive dysphagia involving both solids and liquids and a 5-kg body weight loss. He had no smoking or drinking history. The patient had previously visited 2 different tertiary hospitals, where he underwent neck computed tomography (CT) and magnetic resonance imaging. The neck imaging studies revealed an approximately 3.9-cm mass in the cervical esophagus and multifocal calcified lymph nodes in the lower neck and mediastinum. Ultrasound-guided biopsy of the neck mass showed suspicion of tuberculosis. However, there was no evidence of tuberculosis in the pathologic or microbiologic examinations. During the previous hospital visits, the patient was informed of the findings and the need for empirical treatment of tuberculosis; however, he refused to undergo antituberculosis treatment.

### Diagnostic Assessment

**FIGURE 1:**
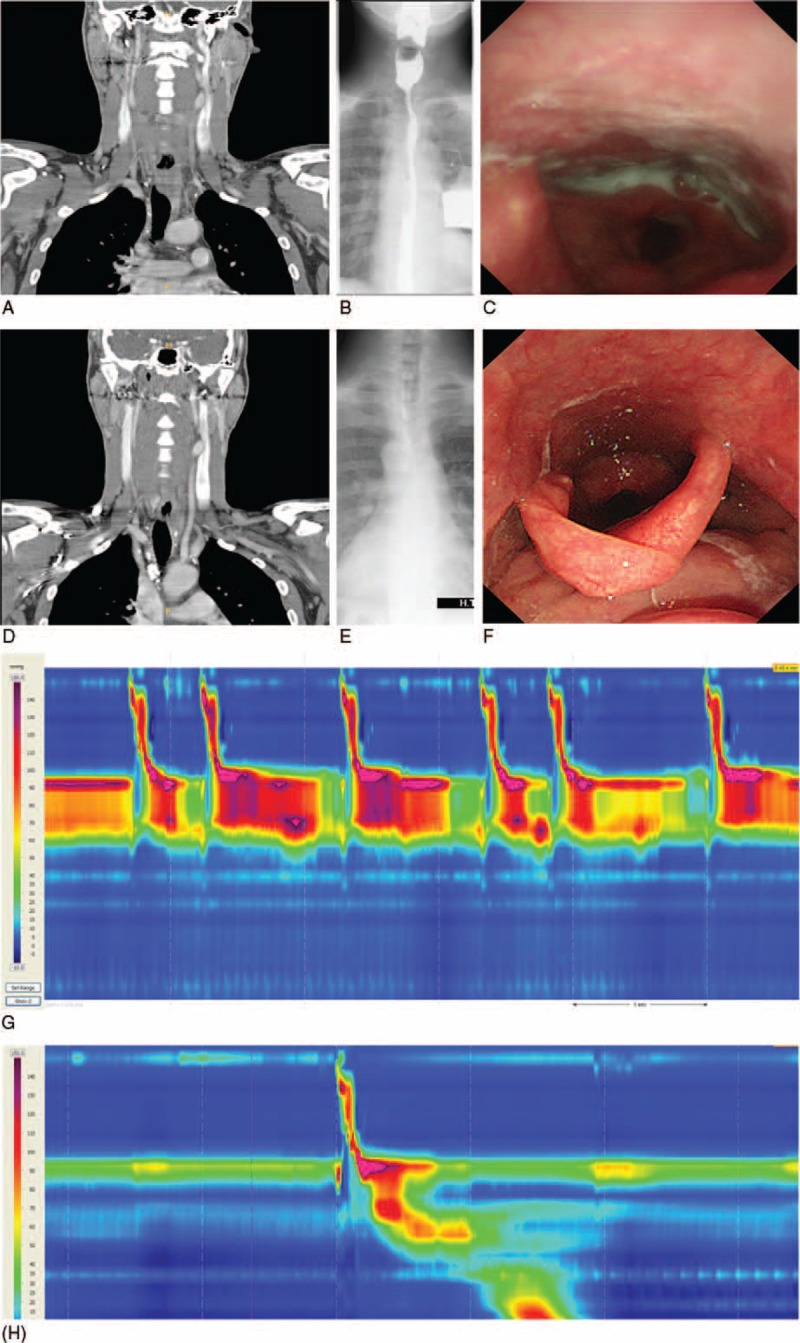
Neck computed tomography, gastrografin esophagography, flexible endoscopic evaluation of swallowing, and high-resolution manometry (A–C, G) before and (D–F, H) after treatment with prednisolone. (A) An approximately 3.9-cm mass in the esophagus causing mild indentation and shifting of the trachea. (B) Dilated pharyngeal cavity with a large amount of residue at both pyriform sinuses. (C) Severe amount of residue at both pyriform recesses. (D) Decreased size of ill-defined mass surrounding lower cervical esophagus and improved indentation of abutting trachea. (E) Near resolution of upper esophageal stricture. (F) No residue at either of the pyriform recesses or the valleculae. (G) Repetitive swallowing and hypercontraction just below the upper esophageal sphincter 11 months after treatment. (H) Complete disappearance of the hypercontraction just above the upper esophageal sphincter and appearance of normal upper esophageal contraction.

Upon presentation to our department, no masses were palpable in the neck, and the physical examination was unremarkable. All laboratory data, including the white blood cell count, platelet count, biochemical parameters, and blood glucose level, were within normal limits. We reviewed the previously performed computed tomography (CT) scan (Figure 1A) and observed the esophageal mass, which had not changed in size and was causing mild indentation and shifting of the trachea. A gastrografin esophagogram revealed a filling defect with a nodular outline involving the cervical esophagus. A large amount of residue was noted at both pyriform sinuses (Figure 1B). Fiberoptic endoscopic evaluation of swallowing (FEES) showed a large amount of residue at both pyriform recesses and relatively less residue at the valleculae, suggesting cricopharyngeal dysphagia (Figure 1C). High-resolution manometry showed repetitive swallowing, hypercontraction just below the upper esophageal sphincter, a hypercontractile portion with a declining pattern related to bolus transit, and a 3.9-cm-long hypercontractile portion (Figure 1G). Upper endoscopy revealed a severe stricture without mucosal change.

**FIGURE 2:**
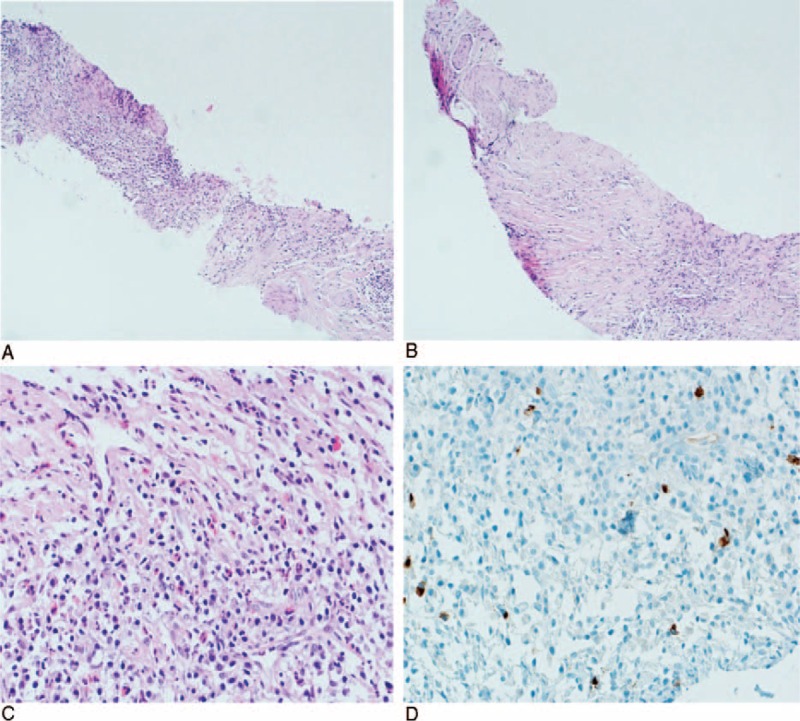
Pathologic findings. Low-power view shows proliferation of spindle cells and small vessels and infiltration of inflammatory cells on a sclerotic background with entrapped (A) blood vessels and (B) nerve fibers (hematoxylin and eosin, ×100). (C) Infiltrating inflammatory cells comprising many plasma cells and eosinophils (hematoxylin and eosin, ×400). (D) Immunohistochemistry exhibits a few IgG4-positive cells.

We also reviewed the pathological sections obtained from the previous ultrasound-guided needle biopsy. Re-examination of the tissue specimens from the esophageal mass revealed proliferation of spindle cells and small vessels and infiltration of inflammatory cells (Figure 2) on a sclerotic background with entrapped blood vessels (Figure 2A) and nerve fibers (Figure 2B). The infiltrating inflammatory cells comprised many plasma cells and eosinophils (Figure 2C). Immunohistochemical examination revealed 12 IgG4-positive cells per high-power field (Figure 2D). The serum IgG4 level was elevated at 264 mg/dL (reference range, <135 mg/dL).

### Therapeutic Intervention, Follow-Up, and Outcomes

Considering all of the above findings, the patient was finally diagnosed with IgG4-RD-associated dysphagia and treated with 40 mg/day prednisolone for 4 weeks with a subsequent 10-mg dose decrease every 2 weeks. He was maintained on steroid therapy at 5 mg/day for 11 months, after which he underwent follow-up neck CT (Figure 1D), gastrografin esophagography (Figure 1E), esophagogastroduodenoscopy, and FEES (Figure 1F). Neck CT showed that the ill-defined mass surrounding the lower cervical esophagus had decreased in size and that the associated tracheal indentation had improved. Esophagography revealed near resolution of the upper esophageal stricture. No residue was present at either of the pyriform recesses or the valleculae on FEES, and high-resolution manometry showed complete disappearance of the hypercontraction just above the upper esophageal sphincter and the appearance of normal upper esophageal contraction (Figure 1H). All study results indicated improvement in the patient's condition. The patient was eventually able to tolerate a solid diet.

## DISCUSSION

Little is known about IgG4-RD-associated dysphagia. To date, only 3 case reports have described IgG4-RD involving the esophagus.^5–7^ (Table 1). The 1st case was mistaken for an esophageal gastrointestinal stromal tumor but was finally diagnosed correctly by surgery. The 2nd case was found to be IgG4-RD after esophagectomy. Interestingly, upper endoscopy with biopsy failed to diagnose the IgG4-RD in either case, even with visibly abnormal mucosal findings in the 2nd case. The most delayed diagnosis was noted in the 3rd case. The present case highlights the diagnostic challenges associated with IgG4-RD in a dysphagic patient. First, the diagnosis is usually delayed due to the extreme rarity of IgG4-RD with esophageal involvement. IgG4-RD has also been recognized recently as an immune-mediated condition. Second, the presentation of IgG4-RD is nonspecific because the symptoms depend on the affected organ. The reported symptoms of esophageal involvement include dysphagia, odynophagia, and weight loss. Endoscopy can reveal simple esophagitis, ulceration, stricture formation, submucosal tumors, or even evidence of malignancy. The duration from the 1st symptom to diagnosis ranges from 11 months to 10 years.^5–7^

**TABLE 1:**
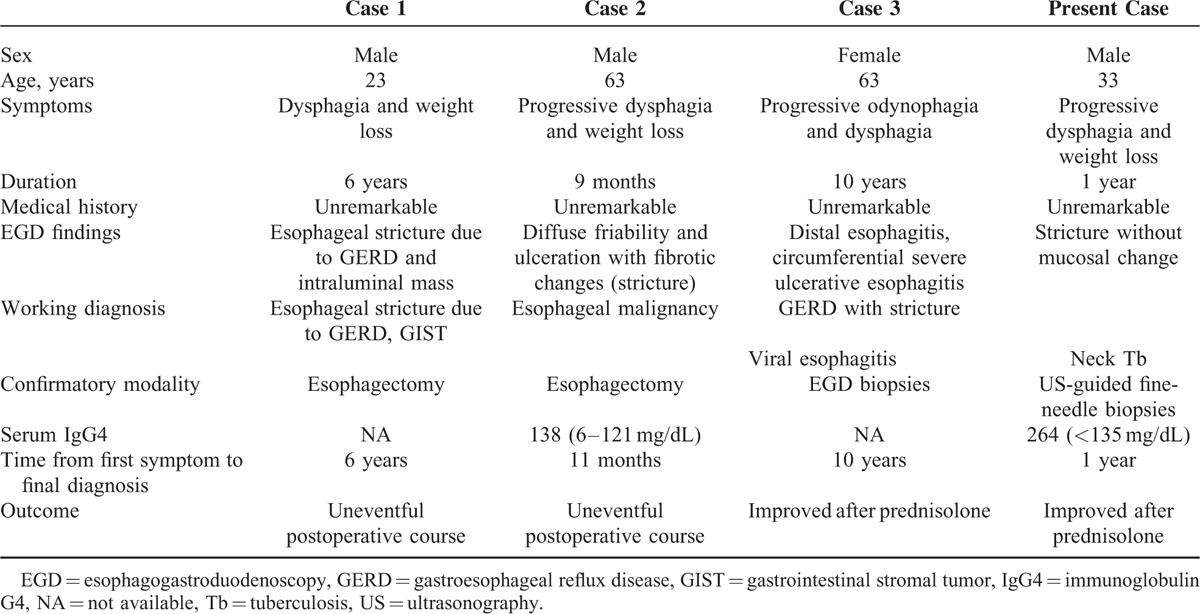
Summary of Previously Published Case Reports of IgG4-Related Esophagitis

Finally, careful interpretation of ultrasound-guided samples can lead to an accurate diagnosis of IgG4-RD. In the present case, the 2 previous hospitals failed to diagnose the IgG4-RD despite the performance of ultrasound-guided needle biopsy. This is because the cytologic findings of IgG4-RD are unfamiliar to many pathologists and gastroenterologists.

In conclusion, clinicians should have a high index of suspicion for IgG4-RD in dysphagic patients presenting with an esophageal stricture, ulcer, or tumor.
